# Application of Argon Plasma Coagulation for Gastrointestinal Angiodysplasia in Children— Experience From a Tertiary Center

**DOI:** 10.3389/fped.2022.867632

**Published:** 2022-04-05

**Authors:** Pai-Jui Yeh, Puo-Hsien Le, Chien-Chang Chen, Hsun-Chin Chao, Ming-Wei Lai

**Affiliations:** ^1^Linkou Branch, Department of Pediatric Gastroenterology, Chang Gung Memorial Hospital, Taoyuan, Taiwan; ^2^Linkou Branch, Department of Gastroenterology and Hepatology, Chang Gung Memorial Hospital, Taoyuan, Taiwan; ^3^Taiwan Association of the Study of Small Intestinal Disease, Taoyuan, Taiwan; ^4^Linkou Branch, Liver Research Center, Chang Gung Memorial Hospital, Taoyuan, Taiwan

**Keywords:** angiodysplasia, Argon plasma coagulation, children, endoscopy, gastrointestinal hemorrhage

## Abstract

**Background:**

Argon plasma coagulation (APC) has been applied in adults to treat various diseases, including vascular lesions in the gastrointestinal (GI) tract. However, angiodysplasia (AD) is an uncommon cause of pediatric GI bleeding, while the experience of treating AD with APC was rarely reported.

**Methods:**

Five children with AD in the GI tract successfully treated with APC were reviewed.

**Results:**

Three of the five patients were girls, and the age at diagnosis ranged from 1.5 months to 10.5 years of age. One patient with gastric AD manifested with tarry stool, and the rest had colonic AD, which caused various degrees of bloody stool. Three patients had evident anemia. All patients received an endoscopic diagnosis, and two had compatible findings in radiographic exams. Each patient underwent one APC treatment session, and none encountered procedure-related complications or re-bleeding.

**Conclusion:**

AD can be an etiology of GI bleeding even in neonates. APC is an effective and safe therapy for symptomatic AD in children.

## Introduction

Argon plasma coagulation (APC) is non-contact electrocoagulation using high-energy monopolar energy to ignite argon gas into a plasma to cauterize and devitalize vascular tissues to achieve hemostasis or debulking tumors (1, 2). It is appreciated for its minimal tissue vaporization and penetration, which limit the risk of perforation. This technique has been applied to treat gastrointestinal (GI) vascular malformation (gastric vascular ectasia, angiodysplasia (AD), arteriovenous malformations, and Dieulafoy’s lesions), Barrett’s esophagus, Zenker’s diverticulum, malignant lesions (ablation, debulking), complex fistula, and even for pulmonary and dermatologic diseases (3–6).

AD is an important etiology of non-variceal GI bleeding, with several synonyms (arteriovenous malformation, angiectasia, and vascular ectasia) and debating hypotheses of pathophysiology (4). It is usually described as abnormal, ectatic, and tortuous vessels within the mucosal or submucosal layers of the GI tract (4). It is the most common vascular malformation of the GI tract, presenting with GI bleeding with variable severity. The incidence increases with age, most common in patients > 60 years of age, making AD an uncommon culprit for pediatric GI bleeding (7). After the diagnosis is established by endoscopy or angiography [primarily *via* computed tomography (CT)], interventions are guided by clinical severity and the characteristics of the lesion. In addition to APC, the treatment of choice, transcatheter angiographic embolization, and pharmacological agents (thalidomide, octreotide) may be incorporated accordingly (4). Surgery may be considered for patients who require massive transfusion or life-threatening bleeding from an identified site. However, recurrent bleeding can occur from lesions missed by pre-operative image, incomplete resection, or new lesions in the GI tract (4, 8).

Pediatric AD of the GI tract was mainly reported in a single case or small series. Thus the clinical experience is insufficient for pediatric gastroenterologists. In addition, literature elaborating on the application of APC in children was even scarcer. Based on this rarity, no therapeutic guidelines for AD are available for both adults and children, except for a few associated sections in the current guidelines for GI bleeding and hereditary hemorrhagic telangiectasia (4, 9–11). In addition, the safety profile for infants is unknown. Herein, we presented five patients with AD in the GI tract treated with APC, including one infant.

## Materials and Methods

### Patients

We retrospectively retrieved five children who received APC for gastrointestinal AD (Table 1). The procedure was performed in collaboration between the pediatric and adult gastroenterology teams over the past 3 years. In our institute, APC has been practiced by adult gastroenterologists for over a decade. The same adult endoscopist executed the procedure to provide a constant APC strategy and technique. Clinical presentations, comorbidities, relevant laboratory data, diagnostic imaging (CT, endoscopy), treatment, and outcomes were reviewed. The diagnosis of AD was defined by the endoscopic findings of typical mucosal lesions (abnormally tortuous or dilated small vessels), with or without concurrent regional change of the alimentary wall in the CT scan. All APC sessions were performed under general anesthesia in our pediatric intensive care unit (ICU), which warranted continuous monitoring during and after the procedure and high-level cautions for complications.

**TABLE 1:** Clinical features of the five patients receiving argon plasma coagulation.

	1	2	3	4	5
Age (year)	1.3	0.1	2.8	10.6	3.2
Sex	M	F	F	F	M
Symptom	Tarry stool	Bloody stool, Diarrhea	Bloody stool	Bloody stool, Abdominal pain	Bloody stool
Hb (g/dL)	6.4	7.2	13.1	13.6	6.4
Diagnosis	EGD	CS, CT	CS, CT	CS	CS
Lesion site	Stomach	D-colon	A-colon, S-colon	40 and 28 cm from anal verge	Cecum
Treatment	APC	APC, Propranolol, Elecare*	APC	APC, propranolol	APC
Outcome	No re-bleeding	No re-bleeding	No re-bleeding	No re-bleeding	No re-bleeding
Follow-up (month)	0.5	19	1	20	8

*A, ascending; APC, argon plasma coagulation; CS, colonoscopy; CT, computed tomography; D, descending; EGD, esophagogastroduodenoscopy; F, female; Hb, hemoglobin; M, male; S, sigmoid.*

* Amino acid-based formula (indication: concurrent allergic proctocolitis).

### Facilities

APC was applied with the VIO^®^ ESU/APC System [VIO ElectroSurgery Unit (ESU) (Model VIO 300 D) equipped with the Argon Plasma Coagulator (Model APC™ 2), ERBE, United States] and the pediatric endoscopic system (Olympus^®^), administered *via* the FiAPC probe (No. 20132-220, flexible, 1500A, 1.5 mm). The PRECISE APC^®^ mode (effect 3) was utilized as the primary setting then adjusted during the procedure. The PRECISE APC^®^ mode delivers uniform tissue effects (8 settings), which is almost independent of the distance from the probe to the tissue. Given that the maximum depth of coagulation is limited to 1.5 mm, it achieves safer depth control and presumably a lower risk of perforation (12). The colonoscopy applied on our case 2 was a pediatric-sized panendoscopy (Olympus^®^ GIF-XP240, insertion tube diameter 7.7 mm, working channel size 2.2 mm) due to her small body size.

## Results

### Case 1

**FIGURE 1:**
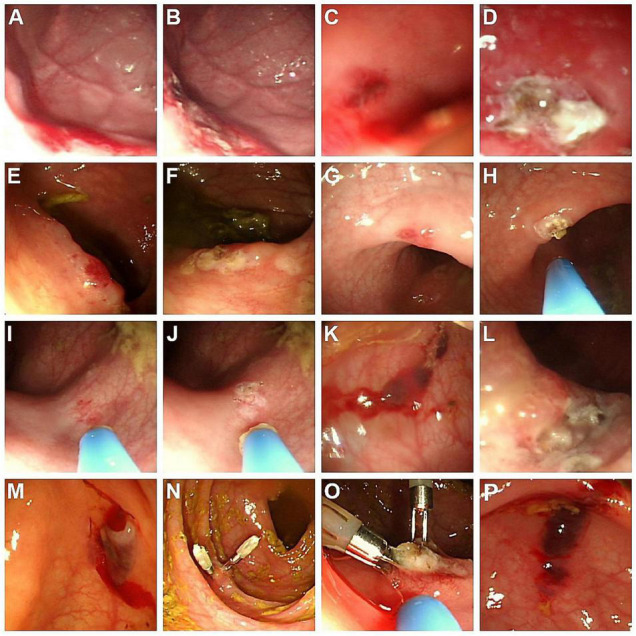
Endoscopic findings of AD in the five cases. **(A)** Case 1: Angiodysplastic lesions at the greater-curvature side of high body and fundus of the stomach; **(B)** lesions of **(A)** treated with APC; **(C)** Case 2: Dark-red papular lesions with oozing in the descending colon; **(D)** lesions of **(C)** treated with APC; **(E,G,I)** Case 3: Several forms of hyperemic, bluish, and irregular AD lesions, located in the ascending and sigmoid colon; **(F,H,J)** lesions of **(E,G,I)**, respectively, treated with APC; **(K)** Case 4: Several bluish vascular lesions with hemorrhage in the descending colon; **(L)** lesions of **(K)** treated with APC; **(M)** Case 5: An oozing angiodysplastic lesion near the cecum; **(N)** lesion of **(M)** with two hemoclips as initial hemostasis; **(O)** lesion of **(N)** treated with APC to manage the residual angiodysplastic part; **(P)** non-bleeding AD lesion in the ascending colon, without APC treatment due to the absence of clinical GI bleeding. AD, angiodysplasia; APC, argon plasma coagulation.

This 1-year-4-month-old boy was admitted due to recurrent tarry stool passage with an interval of one and half a month. He was admitted to a local hospital for 3 days during the first course, and the bleeding ever ceased. However, tarry stool recurred 2 days before admission to our institute, accompanied by a pale appearance. Laboratory tests showed hemoglobin (Hb) 6.4 g/dL, platelet 258,000/μL, international normalized ratio (INR) 1.0, and a normal biochemistry profile. Blood transfusions and intravenous cimetidine were administered. Esophagogastroduodenoscopy (EGD) disclosed mild esophagitis (Los Angeles Classification grade A) and multiple angiodysplastic lesions measured 2–8 mm at the greater curvature side of the high body and fundus of the stomach, covered with fresh blood clots that suggested recent hemorrhage (Figure 1A). Under general anesthesia in the ICU, EGD was repeated for APC therapy (Figure 1B). He was discharged 3 days post-procedure with the recovery of Hb level and remitted tarry stool. No re-bleeding has been documented after that.

### Case 2

**FIGURE 2:**
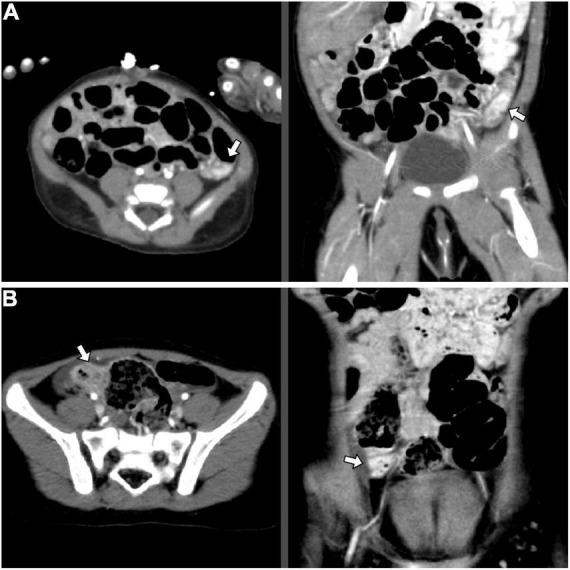
Abdominal CT angiography images indicating corresponding locations of the AD, axial (left) and coronal (right) view: **(A)** Case 2: Wall enhancement of focal segments at the left lower abdomen, suspected of hemangioma or hypervascular lesions in the descending colon (white arrow); **(B)** Case 3: Focal increased wall enhancement at the ascending colon (white arrow). AD, angiodysplasia; CT, computed tomography.

This 1-month-17-day-old girl with failure to thrive (body height 50–85th percentile, body weight < 3rd percentile) presented blood-tinged diarrhea for a week, accompanied by transient low-grade fever and skin rashes. She was initially admitted to a local hospital, where surveys found normocytic anemia (Hb 7.6 g/dL), positive stool occult blood (4+), and negative results for all stool bacterial cultures. The severity did not improve with lactose-free formula feeding, antacid and empiric antibiotics. After referral to our emergency room, she was admitted to our ICU due to impending shock. Laboratory tests showed anemia (Hb 7.2 g/dL), mildly elevated eosinophil percentage (up to 10.4%, absolute count 1,050/μL), thrombocytosis (platelet 825,000/μL), hypoalbuminemia (albumin 1.94 g/dL), normal INR, and normal CRP levels. The abdominal sonography and EGD were unremarkable. Extensively hydrolyzed formula was prescribed, but her bloody diarrhea persisted with continuous anemia requiring blood transfusion. Therefore, colonoscopy was arranged using a pediatric EGD scope, which revealed colitis (rectum to descending colon, the segment reached by the scope) with skipping hyperemic lesions and a dark-red papular lesion in the descending colon (Figure 1C). Abdominal computed tomography (CT) was arranged to evaluate if other vascular lesions were present and reported wall enhancement of rectum and focal segments at the left lower abdomen, suspected of hemangioma or hypervascular lesions in the descending colon (Figure 2A). Three major angiodysplastic lesions with active oozing were treated with APC smoothly (Figure 1D). Colonic biopsies suggested eosinophilic colitis, and cow’s milk protein allergic proctitis was considered. The frequency of diarrhea gradually decreased after changing to an amino acid-based formula (Elecare). However, intermittent bloody stool was still noted. Considering the frequency of colonoscopy and sedation in such a short period (twice in 1 week), and inaccessible proximal colonic segments, propranolol was prescribed. Her bloody stool lessened following the use of propranolol (oral form, 1 mg/kg/day after gradual titration) without further need for blood transfusion. Subsequent laboratory tests affirmed the correction of anemia and hypoalbuminemia. During outpatient follow-up, her formula was stepwise shifted to extensively hydrolyzed formula (Alfare), with a daily stooling of 1–5 times and continuous weight gain (3rd to 10th percentile). No more gross hematochezia was noted, and propranolol was gradually tapered off. In the subsequent 1.5 years, she was only admitted once for bacterial enterocolitis.

### Case 3

This 2-year-9-month-old girl came to our clinic with the presentation of intermittent bloody stool for 2 months. The blood streaks and mucus seemed to be mixed in the feces and occurred 1–2 times weekly without accompanied fever, abdominal pain, vomiting, constipation, diarrhea, or any sign of systemic bleeding tendency. Laboratory tests showed normal hemogram, coagulation profile, and biochemistries. Stool analysis, stool cultures, and abdominal sonography were all negative. Colonoscopy demonstrated at least two hyperemic, bluish, and irregular lesions, measured 1–5 mm, located in the ascending and sigmoid colon (Figures 1E,G,I). Abdominal CT angiography reported focal increased wall enhancement at the ascending and sigmoid colon, compatible with the characteristics of angiodysplasia (Figure 2B). Hence, APC was performed to manage multiple angiodysplasia lesions (Figures 1F,H,J). After discharge, no anal bleeding recurred.

### Case 4

This 10-year-7-month-old girl developed intermittent abdominal pain for around a month, which was not improved with regular treatment for gastroenteritis at a local hospital. There was no fever, vomiting, or diarrhea, but blood-tinged stool a few times. Physical examination found no signs of anemia or acute abdomen. However, her pain did not resolve with hydration, antacid, and prokinetics therapies. Laboratory tests, stool analysis, and abdominal sonography reported no significant findings. EGD showed reflux esophagitis and mild gastritis. Colonoscopy disclosed several bluish vascular lesions with hemorrhage noted approximately 40 cm from the anal verge (Figure 1K). APC was applied for two major sites, including a 1.5 cm hemangioma-like lesion at 40 cm and a 0.5 cm angioectasia at 28 cm (Figure 1L). A short course of oral propranolol was prescribed after the procedure, and she recovered smoothly with a resolution of both abdominal pain and hematochezia.

### Case 5

This 3-year-2-month-old boy had two admissions 6 months apart due to maroon-colored stool. Initially, he developed painless bloody (maroon red) stool for 2 days without fever, emesis, or bowel habit change. Physical examination, including digital rectal exam, disclosed no specific finding. Laboratory test (the first admission) found mild normocytic anemia (Hb 10.4 g/dL), normal INR, platelet count, and CRP level. Stool analysis showed occult blood (3+). Abdominal sonography, plain films, and Meckel’s scan were unremarkable. He had no more hematochezia in the hospital and was discharged. However, maroon-colored stool recurred 6 months later in a similar painless and afebrile pattern. The second Meckel’s scan still yielded negative findings. The initial Hb level (11.8 g/dL) was normal but dropped 2 days later after once-daily episodes of hematochezia (6.9 g/dL). Despite blood transfusion, the Hb level worsened (6.4 g/dL) after a further bloody stool episode, accompanied with palor, tachycardia, and borderline hypotension. Blood transfusion continued, and he was transferred to ICU, yet the emergent CT angiography did not reveal a significant contrast extravasation site. EGD showed multiple shallow duodenal ulcers without the stigma of bleeding. Colonoscopy disclosed an active oozing angiodysplasia near the cecum, and hemoclipping was performed with successful hemostasis (Figures 1M,N). A session of APC was performed 3 days later to obliterate the lesion (Figure 1O). He regained stable vital signs after hemoclipping and started intake a day after the APC procedure, and his Hb levels also improved steadily without transfusion. No more bloody stool was observed in the subsequent outpatient follow-ups.

## Discussion

This study presented five children manifesting with a variety of GI hemorrhages, diagnosed as gastrointestinal AD, and treated with APC. Generally, the therapeutic efficacy of APC was acceptable without requiring a second session or procedure-associated complication. Best to our knowledge, case 2 could be the youngest infant uneventfully treated with colonic APC.

The incidence of gastrointestinal AD in children was unclear due to its rarity and low diagnostic awareness. Therefore, AD is not a common differential diagnosis for GI bleeding in children; in addition, endoscopy may be waived due to invasiveness for children, especially infants (7, 13). Delayed or underdiagnosis was possible and has ever been stated in some studies. In a series focusing on pediatric colonic AD, the diagnosis was deferred by an average of 2.9 years (range: 5 months to 7 years); another series observed a range of 1 week to 11 years between the bleeding onset to the diagnosis (7, 13). A few cases of pediatric AD with substantial diagnostic challenges have been reported, such as transfusion-dependent pan-colonic lesions mimicking inflammatory bowel disease, jejunal AD with profuse anemia requiring laparotomy surgeries, and diffuse microscopic AD identified by intraoperative transluminal endoscopy (14–16). Our series only included patients treated with APC; thus, it might not represent the whole spectrum. Nonetheless, case 2 was complicated with concurrent eosinophilic colitis, case 3 encountered a lag of 2 months before AD diagnosis, delayed by ineffective treatment as enterocolitis at a local clinic, and case 5 with massive maroon-colored painless bleeding mimicking the presentation of Meckel’s diverticulum. Meanwhile, we occasionally encountered non-bleeding, “incidental” AD in endoscopies. For instance, a 12-year-old boy had an AD in the ascending colon during a coloscopic investigation for prolonged abdominal pain, yet intervention (such as APC) was not administered since there was neither history of GI bleeding nor anemia (Hb 15.2 g/dL) (Figure 1P). As suggested in the literature, management of AD should be tailored according to the individual’s clinical factors, including the characteristic of the lesion and the history of occult/overt GI bleeding. Treatment for these incidental lesions is not considered mandatory, although more comprehensive evidence is needed (4, 8).

A few distinct features between children and adults are noteworthy. In adults, the incidence generally inclines with age, but the diagnostic age for children seems variable, even with occurrence in infants. Our five cases ranged from 1.5 months to 10.5 years of age, corresponding to the ranges in the prior two studies: 1 week to 11 years (average, 2.3 years) and 1 month to 17 years (median 6.2 years, average 7.1 years), respectively (7, 13). Second, our cases did not carry any specific underlying AD-associated diseases in adults (or rarely in children), such as aortic stenosis, Von Willebrand disease, renal failure, collagen tissue disease, Klippel Trenaunay syndrome, and blue rubber bleb nevus syndrome (4, 17, 18). Third, the right-sided predominance of colonic lesions in adults seems less definite in children since only two patients among four colonic AD involved the ascending colon in this series. Chuang et al. revealed AD predominantly involved ascending colon and terminal ileum, yet the other four reports claimed AD majorly on the left-sided colon (7, 13, 17, 19, 20). With these disparities, the pathophysiology of AD in children may be distinct from Boley’s theory (aging-related chronic low-grade obstruction of submucosal veins) hypothesized for adults’ AD (4, 7, 13).

APC is widely applied for treating AD and other GI lesions with or without hemorrhage in adults, but clinical experience in children is scarce. In our series, the therapeutic efficacy and safety profile were generally acceptable; there was neither overt re-bleeding nor complication, even for treating infantile colonic AD. In addition to the accommodation of smaller endoscopy, our endoscopists adopted the PRECISE mode of APC, which features an automatic adjustment of its continuously delivered energy and a more constant tissue effect independent of the distance between the target and the probe. Each burst is controlled short and modified meticulously according to the gross tissue reaction. Lee et al. depicted a 10-day-old neonate with a huge gastric hemangioma refractory to conventional hemostasis and was eventually staunched after two sessions of APC; this was the youngest case treated with APC in literature, demonstrating the applicability in such a vulnerable group of patients (21). Another report described a boy with renal failure who developed hematochezia due to colonic AD and recovered after APC and octreotide (20). Khan et al. described the largest and most comprehensive series describing 23 APC procedures for GI lesions in children: successful hemostasis rate and re-bleeding rate were 66.7% (8/12) and 25% (3/12) (22). Another series of three children with gastric AD reported the need for a second or third APC procedure for residual lesion or re-bleeding in two (23). Reviews on adult series have reported the re-bleeding rates ranging from 11 to 19% and 2 to 10% for small intestine and colon lesions, respectively (4, 24). Procedure-related complications, including submucosal argon gas and scar formation, occurred in 15 and 1.7% of children and adults, respectively (22, 24). Severe complication, such as colonic gas explosion and intestinal perforation, has not been reported in children. Compared to APC for adults patients, several issues are challenging when performing APC in children, including limited endoscopic instruments (tube diameter, working channel, APC probe), less efficacious bowel cleansing with a higher risk of the explosive event, thinner mural thickness, and a lack of standard protocol of APC setting (22). Like other interventional endoscopic therapies in children, gentle care, and higher caution are critical for a safe procedure.

The study’s limitations included the small case number, variable patient characteristics (lesion sites, underlying diseases, severity), potential diagnostic biases from the identification and interpretation of angiodysplastic lesions by the endoscopists, and a heterogeneous body of prior pediatric literature to compare with. The therapeutic outcomes in different alimentary sites and various APC modes were not comprehensively analyzed due to the lack of details in prior reports. However, the present and previous series demonstrated the potential of APC as an efficient and safe approach for symptomatic pediatric AD, even for neonates, under the cooperation of pediatric and adult endoscopists. Nonetheless, up to one-third of patients underwent surgeries for intestinal AD due to recurrent bleeding or unstable vital signs in a pediatric series (7). Given the advance in interventional endoscopy, APC might be a valuable alternative treatment to surgery in pediatric AD in the GI tract.

## Conclusion

Angiodysplasia (AD) is an uncommon cause of gastrointestinal bleeding in children. Argon plasma coagulation (APC) is a therapeutic option for such kids. This study demonstrated the applicability, safety profile, and potential of APC in treating pediatric gastrointestinal AD, even for infants.

## Data Availability Statement

The original contributions presented in the study are included in the article/supplementary material, further inquiries can be directed to the corresponding author/s.

## Ethics Statement

Ethical review and approval was not required for the study on human participants in accordance with the local legislation and institutional requirements. Written informed consent from the participants’ legal guardian/next of kin was not required to participate in this study in accordance with the national legislation and the institutional requirements.

## Author Contributions

P-JY, P-HL, and M-WL: study conception and design and analysis and data interpretation. P-JY, P-HL, H-CC, C-CC, and M-WL: data acquisition. P-JY: drafting of the manuscript. M-WL: critical revision. All authors: final approval.

## Conflict of Interest

The authors declare that the research was conducted in the absence of any commercial or financial relationships that could be construed as a potential conflict of interest.

## Publisher’s Note

All claims expressed in this article are solely those of the authors and do not necessarily represent those of their affiliated organizations, or those of the publisher, the editors and the reviewers. Any product that may be evaluated in this article, or claim that may be made by its manufacturer, is not guaranteed or endorsed by the publisher.
